# Case Report: A Missense Mutation of *KIT* in Hyperpigmentation and Lentigines Unassociated With Systemic Disorders: Report of a Chinese Pedigree and a Literature Review

**DOI:** 10.3389/fmed.2022.847382

**Published:** 2022-05-25

**Authors:** Lu Yang, Yuehua Liu, Tao Wang

**Affiliations:** State Key Laboratory of Complex Severe and Rare Diseases, Department of Dermatology, Peking Union Medical College Hospital, Chinese Academy of Medical Sciences and Peking Union Medical College, National Clinical Research Center for Dermatologic and Immunologic Diseases, Beijing, China

**Keywords:** KIT, hyperpigmentation, generalized lentigines, missense mutation, hotspot

## Abstract

**Background:**

*KIT* is a proto-oncogene that is involved in the proliferation, survival, and regulation of melanocytes, mast cells, and the interstitial cells of Cajal. Mutations of *KIT* have been reported to be associated with hyperpigmentation and lentigines, mastocytosis, and gastrointestinal stromal tumors (GISTs). Some hotspot mutations of *KIT* have been reported to be associated with mastocytosis and GISTs, while the relationship between *KIT* mutations and hyperpigmentation and lentigines has not been fully elucidated.

**Methods:**

In this study, we presented a three-generation Chinese pedigree with progressive hyperpigmentation and generalized lentigines inherited in an autosomal dominant pattern. High-throughput sequencing was performed to capture genetic variations in peripheral blood samples of the proband. Also, Sanger sequencing was performed to further verify the result. We also reviewed previous literature on *KIT* mutations with hyperpigmentation and lentigines.

**Results:**

A missense mutation of the *KIT* gene was identified: c. 2485G > C, which was co-segregated in the proband and his insulted father. Germline *KIT* mutations presenting as generalized hyperpigmentation and lentigines without systemic disorders are rare, with only two reports of c. 2485G > C mutation associated with this phenotype in previous literature.

**Conclusion:**

Our pedigree, together with those two reports, indicates a possible phenotype-genotype correlation of this germline *KIT* mutation, which might be helpful for genetic counseling, further functional segregation of KIT, and design of targeted therapy in the future.

## Introduction

Lentigines are usually a benign proliferation of epidermal melanocytes, which are commonly seen in sun-exposed areas. However, numerous or generalized lentigines might be an indicator of systemic disorders, including Peutz-Jeghers syndrome, Carney complex, LEOPARD syndrome, Dowling-Degos disease, *PTEN* hamartoma tumor syndrome, and familial progressive hyperpigmentation with or without hypopigmentation (1).

*KIT* (OMIM:164920) is a proto-oncogene, which encodes a type III receptor tyrosine kinase for stem cell factors. It plays an important role in the development and regulation of melanocytes, mast cells, germ cells, the interstitial cells of Cajal, and hematopoietic progenitor cells (2, 3). *KIT* mutations are reported to be associated with a variety of cutaneous disorders, including piebaldism, mastocytosis, hyperpigmentation, and lentigines (1, 4–8). They are also involved in malignancies including testicular germ cell tumor, acute myelogenous leukemia, gastrointestinal stromal tumor (GIST), and melanomas (2, 6, 9). Some hotspots of somatic and germline mutations of *KIT* have been found in GIST and mastocytosis, but not in hyperpigmentation and lentigines (2).

There are two reports of a missense mutation at the kinase activation loop of *KIT* (c. 2485G > C) that is associated with hyperpigmentation and lentigines without GIST or mastocytosis (1, 5). In this study, we reported a Chinese pedigree with progressive hyperpigmentation and generalized lentigines in which the same missense mutation of *KIT* was confirmed. We also reviewed the literature and discussed a possible phenotype-genotype correlation in this entity.

## Materials and Methods

### Pedigree and Subjects

We studied a three-generation Chinese pedigree presented as hyperpigmentation and generalized lentigines. Peripheral blood specimens were collected for genetic analysis. Written informed consent was obtained from this patient and his parents for skin biopsy, mutation screening, and publication of their images or data included in this article. The study was approved by the Institutional Review Board.

### Mutation Screening and Sequencing

The DNA library was constructed by hybridization capture from the genomic DNA extracted from the peripheral blood samples after fragmentation, splicing, amplification, and purification, and was detected by a high-throughput sequencing platform (mean sequencing depth, 130.19 ×). The original sequencing data were compared with the UCSC hg19 human reference genome sequence and further filtered out with synonymous variants or small non-frame shift InDel variants in repeat regions. The remaining data were analyzed according to the inheritance patterns and clinical phenotypes. Candidate variations were further verified by Sanger sequencing. The pathogenicity of genetic variation was classified according to ACMG guidelines (10).

## Results

**Figure 1:**
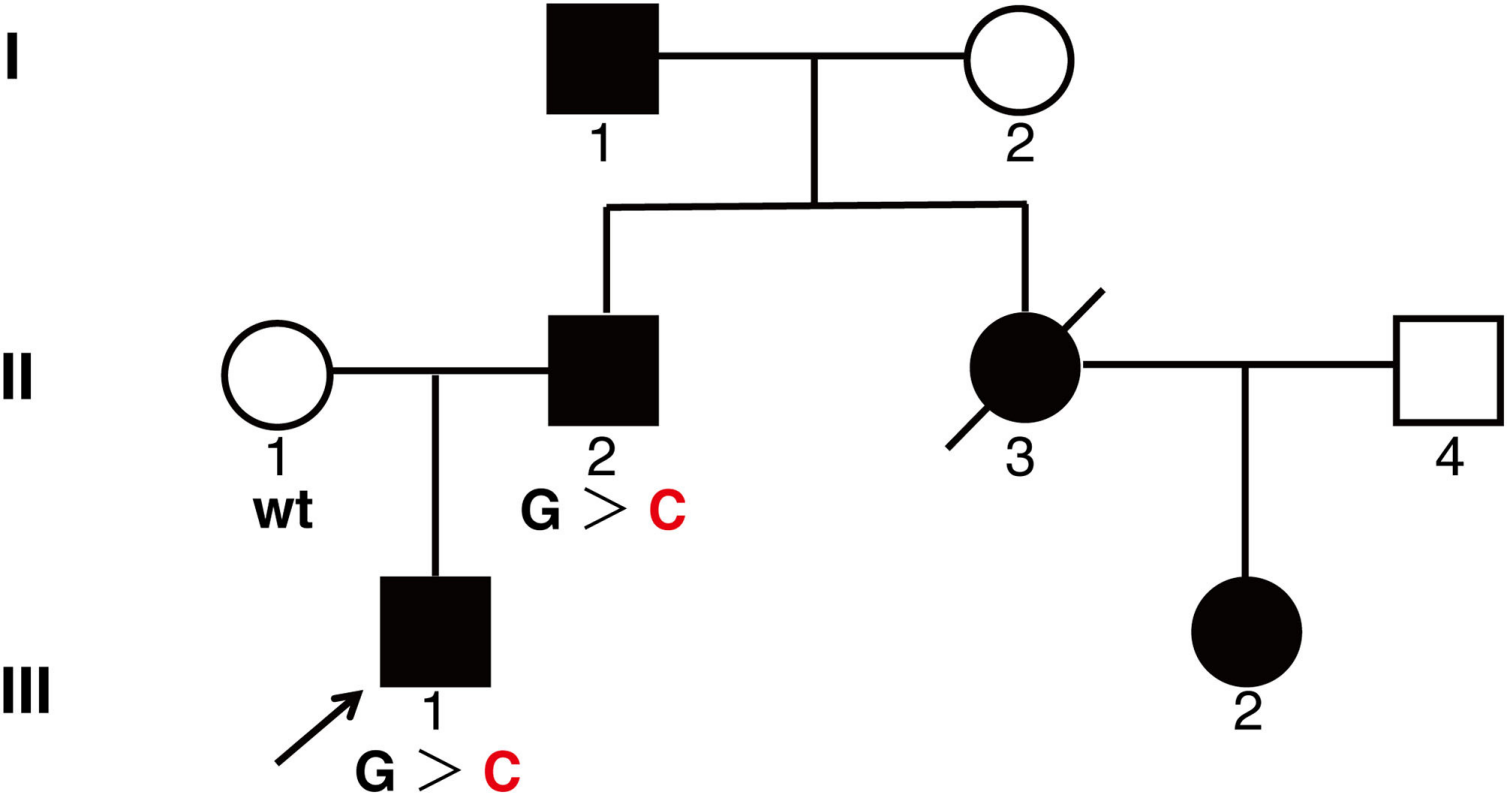
A pedigree showing phenotypes of the family. Three family members were genotyped for *KIT* mutation. Affected men and women are indicated by filled squares and circles, respectively. A crossed symbol indicated a deceased individual due to urenia. An arrow head indicates the proband. wt, wild type; G > C, heterozygote of c.2485G > C.

**Figure 2:**
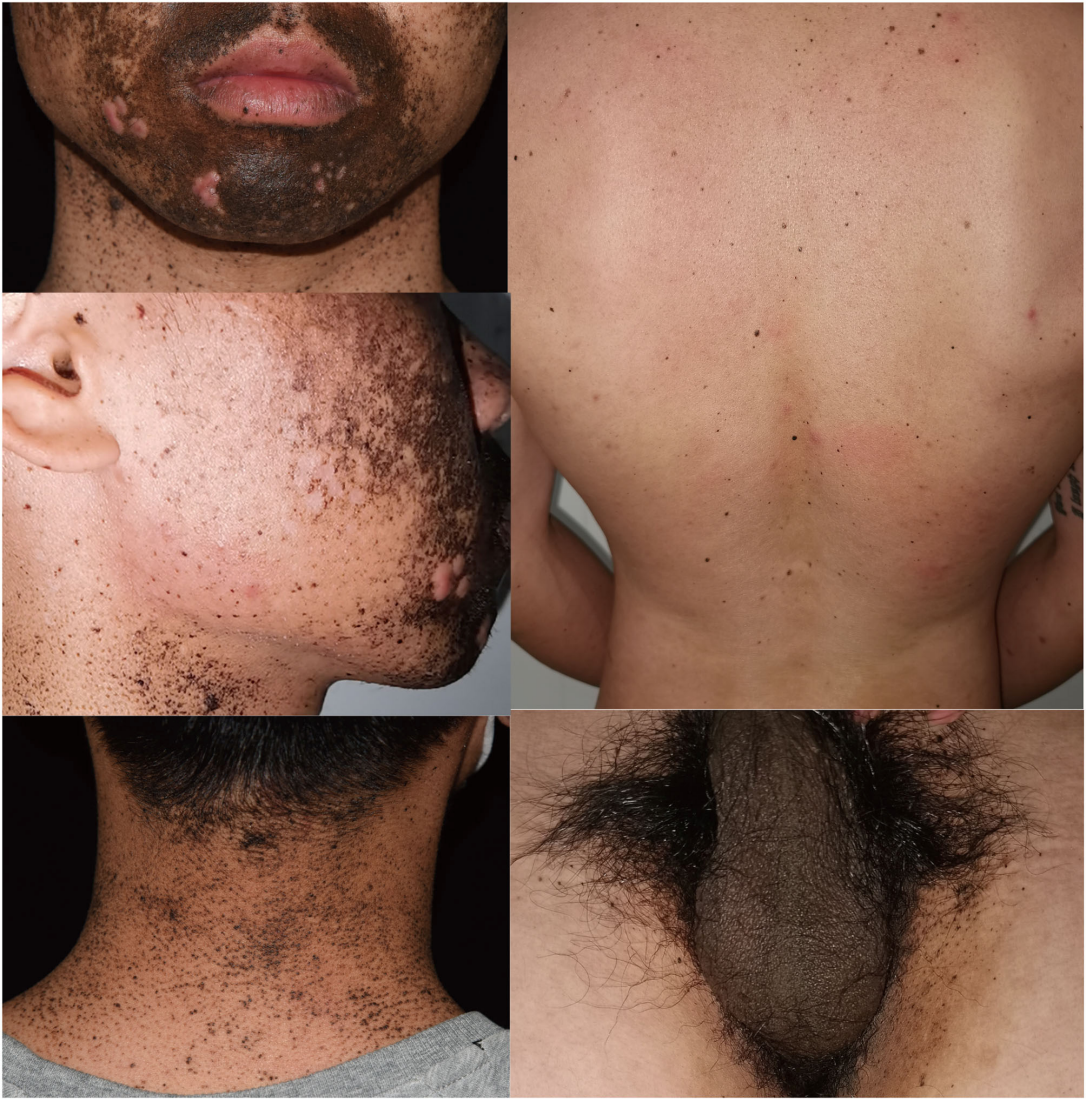
Clinical presentation of the proband showing hyperpigmentation and multiple lentigines on face, neck, back, and inguinal areas.

A 26-year-old man came to our department complaining of progressive hyperpigmentation and generalized lentigines over his body for 18 years. The patient had a family history that his father, grandfather, aunt, and cousin had similar symptoms (Figure 1). He was otherwise healthy without abdominal pain or mass, dysphagia, and weight loss. The patient and his father underwent annual physical examination without abnormal routine blood and urine test, liver, and renal function test, and abdominal CT scans. His grandfather lived a healthy longevous life without any obvious systemic disorders. Physical examination revealed hyperpigmentation with a dark brown spot and lentiginous-like lesions on his face, neck, vermillion of the lip, inguinal region, natal cleft, hands, and feet (Figure 2). Similar lesions were scattered on his trunk and extremities. The oral cavity was not involved. There was no tenderness or palpable mass of the abdomen.

**Figure 3:**
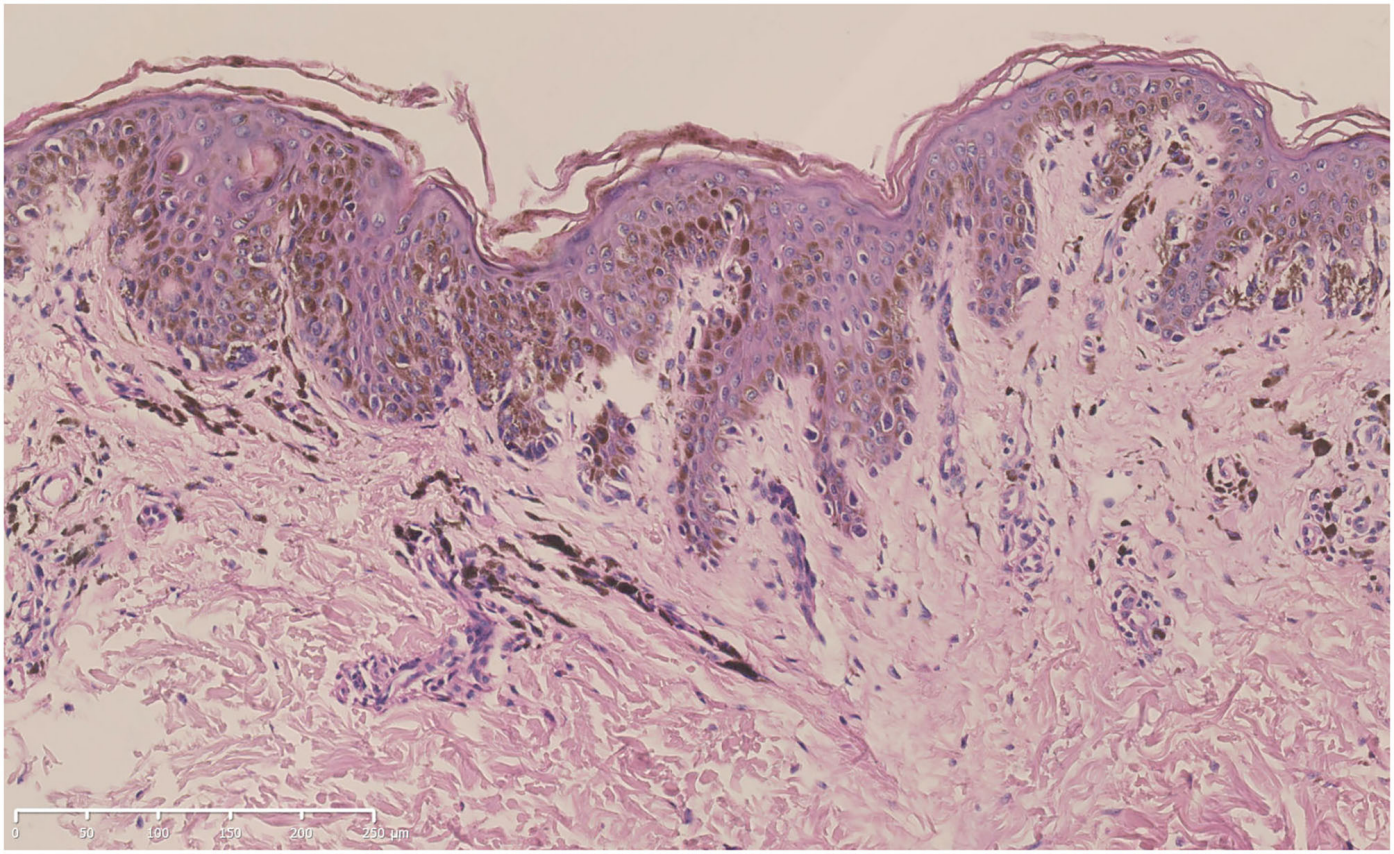
Hematoxylin-eosin staining of the biopsy specimen from the proband (bar = 250 μ*m*). Increased melanin production from the basal layer to the spinous layer of the epidermis, lentiginous hyperplasia of melanocytes along the basal layer, and melanophages in the superficial dermis could be seen.

A skin biopsy was taken from one of the lentigines after obtaining written informed consent. Histologic examination revealed increased melanin production from the basal layer to the spinous layer of the epidermis. Lentiginous hyperplasia of melanocytes along the basal layer and melanophages in the superficial dermis could be observed (Figure 3).

An inherited disorder was highly suspected. After obtaining informed consent, peripheral blood samples were obtained for further genetic analysis. High-throughput sequencing revealed c. 2485G > C mutation of *KIT* in the proband (III:1), which was a likely pathogenic mutation according to the ACMG standards and guidelines (10). Sanger sequencing was performed and verified the heterozygous c. 2485G > C mutation both in the proband (III:1) and his insulted father (II:2), while his mother did not carry the mutation.

## Discussion

*KIT*, located on human chromosome 4q12, encodes a type III receptor tyrosine kinase composed of an extracellular ligand-binding domain, a transmembrane domain, a juxta-membrane domain, and an intracellular kinase domain. Stimulation by stem cell factor leads to its dimerization, followed by activation of its intrinsic kinase activity and phosphorylation of specific tyrosine residues in the intracellular domain. These residues could act as a docking site to recruit downstream signaling molecules (2, 3). Although KIT is involved in lineage commitment and regulation of interstitial cells of Cajal, mast cells, and melanocytes, different *KIT* mutations may not induce the simultaneous deregulation of all three types of cells. Somatic D816V mutation of *KIT* in exon 17 is the most common mutation in mastocytosis, while genetic variations in exons 9 and 11 are most commonly seen in GISTs. These hotspot sites suggest a possible phenotype-genotype correlation in *KIT*, although the underlying mechanism is not clarified (2).

In contrast to mutations in mastocytosis and GISTs, mutations of *KIT* in melanomas have been found throughout the coding regions, with increased frequency in the juxta-membrane domain and tyrosine kinase domains (3). Somatic *KIT* mutations have been reported in both primary and metastatic melanomas, especially in triple-negative melanomas lacking *BRAF, NRAS, or NF* mutations, accounting for 6–20% of melanomas arising on mucosal, acral, and chronically sun-damaged sites (3, 9, 11)/ In a recent large retrospective study of a Chinese patient with melanoma, the frequency of *KIT* mutation was 9.4%, which was comparable with that of the Caucasian population. While a more enriched mutation hotspot region was observed in the cohort, with *KIT* mutations most frequently found in exon 11 (72.3%) (12). However, the study was based in a single center. A large population-based study is needed to further draw the mutation landscape of *KIT* in the future. Until now, there have been 36 *KIT* mutations reported to be associated with melanomas. However, the oncogenicity of these mutations is not fully elucidated, with only 18 of them predicated to be oncogenic or likely oncogenic based on an *in vitro* experiment and some prediction modalities, probably acting through the regulation in MAPK/MEK, PI3K/AKT, and JAK/STAT pathways (3, 11).

Apart from somatic mutations of *KIT* in melanomas, germline *KIT* mutations are reported to be associated with other pigmented disorders, often accompanied by GIST or mastocytosis (1, 2, 4–7). However, two independent groups have recently revealed a new missense mutation of *KIT* in patients with hyperpigmentation and lentigines without systemic disorders. T. Takeichi et al. reported the c. 2485G > C mutation in exon 17 of *KIT* in a Japanese pedigree that manifested as progressive hyperpigmentation and lentigines unassociated with any other familiar systemic disease (5). Almost at the same time, Alain K. et al. revealed the same mutation in a 6-year-old girl who presented with atypical lentiginosis and hyperpigmentation without systemic disorders (1).

In this report, we examined a Chinese pedigree presented with progressive hyperpigmentation and generalized lentigines, carrying the same germline mutation of *KIT*, suggesting its role in the regulation of the proliferation and melanin production of melanocytes. This missense mutation would lead to an amino acid substitution from alanine to proline at position 829 (A829P) on the kinase activation loop of KIT, generating a unique sequence “Pro-Arg-Leu-Pro,” which was postulated to serve as an Src homology 3 (SH3) domain-binding motif and activate the MAPK signaling cascade (2, 5). It has been reported that a secondary somatic A829P mutation of *KIT* was associated with acquired drug resistance during long-term treatment with imatinib mesylate in GISTs (13, 14). Similarly, *in vitro* study has shown that a secondary A829P mutation of *KIT* in the melanoma cell line would lead to the acquired resistance of both imatinib mesylate and sunitinib, while remaining responsive to dasatinib and nilotinib, with unknown mechanisms (9). These findings indicated that the A829P mutation of *KIT* might disturb the interaction interface of specific drugs with the kinase. Clinicians should keep in mind such differences when considering receptor tyrosine kinase inhibitors as a treatment option for patients with hyperpigmentation and lentigines carrying A829P mutation of *KIT*.

It is worth noting that, until now, all the reports of primary germline c. 2485G > C mutation of *KIT*, including our pedigree, have been presented as hyperpigmentation and lentigines without prominent systemic disorders. This indicates a possible phenotype and genotype correlation of this missense mutation, which would be an ideal model to study the proliferation, survival, and melanogenesis of melanocytes. However, considering the association of somatic A829P mutation of *KIT* with tumors, including GISTs and melanomas reported in previous literature, a close follow-up of the patients should be carried out for surveillance of possible atypical hyperplasia, melanomas, or other types of tumors in the future.

## Data Availability Statement

The datasets presented in this study can be found in online repositories. The names of the repository/repositories and accession number(s) can be found below: GenBank, Accession Number: OM304357.

## Ethics Statement

Written informed consent was obtained from the patients for the publication of potentially identifiable data and images.

## Author Contributions

YL and TW contributed to conception and design of the study. TW collected clinical and histologic data. YL organised the data, constructed the figures, and wrote the first draft of the manuscript. All authors contributed to manuscript revision, read, and approved the submitted version.

## Conflict of Interest

The authors declare that the research was conducted in the absence of any commercial or financial relationships that could be construed as a potential conflict of interest.

## Publisher's Note

All claims expressed in this article are solely those of the authors and do not necessarily represent those of their affiliated organizations, or those of the publisher, the editors and the reviewers. Any product that may be evaluated in this article, or claim that may be made by its manufacturer, is not guaranteed or endorsed by the publisher.
